# High Gamma Band EEG Closely Related to Emotion: Evidence From Functional Network

**DOI:** 10.3389/fnhum.2020.00089

**Published:** 2020-03-24

**Authors:** Kai Yang, Li Tong, Jun Shu, Ning Zhuang, Bin Yan, Ying Zeng

**Affiliations:** ^1^PLA Strategy Support Force Information Engineering University, Zhengzhou, China; ^2^MOE Key Lab for Neuroinformation, The Clinical Hospital of Chengdu Brain Science Institute, University of Electronic Science and Technology of China, Chengdu, China

**Keywords:** EEG, emotion, high gamma band, functional network, fusion feature

## Abstract

High-frequency electroencephalography (EEG) signals play an important role in research on human emotions. However, the different network patterns under different emotional states in the high gamma band (50–80 Hz) remain unclear. In this paper, we investigate different emotional states using functional network analysis on various frequency bands. We constructed multiple functional networks on different frequency bands and performed functional network analysis and time–frequency analysis on these frequency bands to determine the significant features that represent different emotional states. Furthermore, we verified the effectiveness of these features by using them in emotion recognition. Our experimental results revealed that the network connections in the high gamma band with significant differences among the positive, neutral, and negative emotional states were much denser than the network connections in the other frequency bands. The connections mainly occurred in the left prefrontal, left temporal, parietal, and occipital regions. Moreover, long-distance connections with significant differences among the emotional states were observed in the high frequency bands, particularly in the high gamma band. Additionally, high gamma band fusion features derived from the global efficiency, network connections, and differential entropies achieved the highest classification accuracies for both our dataset and the public dataset. These results are consistent with literature and provide further evidence that high gamma band EEG signals are more sensitive and effective than the EEG signals in other frequency bands in studying human affective perception.

## Introduction

Emotions play an important role in our daily life; they are involved in cognitive processes such as memory, learning, and decision-making (Zhang et al., 2015). The studies on neuroscience, psychology, and cognitive science show that physiological signals can reflect human emotional states (Dan, 2012). Of all physiological signals, electroencephalography (EEG) signals have the advantage of high temporal resolution, and EEG signals are difficult to conceal; therefore, they have been widely used in emotion recognition (Li et al., 2018).

Evidently, brain activities are usually accompanied by changes in the EEG frequency. Previous studies have reported that alertness and motor imagery are related to low-frequency EEG signals, whereas attention, memory, and emotions are typically correlated with high-frequency EEG signals (Miltner et al., 1999; Balconi and Lucchiari, 2008; Li and Lu, 2009). In recent years, researchers have suggested connections between high-frequency-band activities and emotions. High-frequency-band (>30 Hz) activities reflect the characteristics of emotional integration (Matsumoto et al., 2010); in particular, high gamma band (50–70 Hz) plays an important role in the cognitive control of emotions (Tang et al., 2011). Certain studies have examined that high frequency responses to affective pictures; most of these studies described enhanced responses to emotional stimulus, particularly against negative stimuli (Güntekin and Basar, 2007; Julie and Scott, 2009; Martini et al., 2012). A similar response to affective pictures in high frequency bands has also been observed in studies using invasive intracranial EEG signals; researchers found that emotional pictures are associated with replicable modulations of broadband high gamma band (70–150 Hz) invasive intracranial EEG signals. Unpleasant stimuli elicit a stronger response in the lateral–occipital and occipital–temporal areas than neutral stimuli, and pleasant pictures elicit stronger responses than other stimuli in the high gamma band (Boucher et al., 2014).

Although the high frequency component of EEG signals has been investigated in emotion processing, most of these pioneering studies have focused on the activities of the local brain areas involved in affective perception. The human brain is a complex system; even a simple brain activity involves interactions among various brain regions (Straaten and Stam, 2013; Li et al., 2019). Emotion is a high-level cognitive function, and the processing of emotions requires the cooperation of multiple brain regions (Bassett and Bullmore, 2015). Brain networks (BNs) that can describe the relationships and information interactions among the various brain regions have been widely used in studying the brain activity mechanism (Bassett and Gazzaniga, 2011; Straaten and Stam, 2013). Recently, various studies on emotions have examined emotional specificity using EEG-based functional brain connectivity. Hossein et al. proposed that exposure to joyful stimuli elicits stronger connectivity in the frontal inter/intra-hemispheric regions than the connectivity elicited via exposure to neutral or melancholic stimuli (Hossein and Sahar, 2016). Zhang et al. found that the prefrontal region plays the most important role in emotion processing and interacts with almost all other regions (Zhang et al., 2017). Furthermore, Li et al. reported that connections with significant differences between the negative and neutral valences in the gamma band (30–48 Hz) are much denser than the connections in the beta band (12–30 Hz). The connections mainly occur in the right frontal and parietal–occipital lobes (Li et al., 2019). These studies demonstrate that EEG-based functional connectivity can effectively reflect the specificity of different emotional states.

As discussed above, EEG-based functional BNs can depict the information interaction between the brain regions during emotion processing. However, few studies have exploited network connection patterns under different emotional states in the high gamma band (50–80 Hz). We believe that this research is worth pursuing because high gamma band activities are sensitive to emotion processing, and high gamma band network connections may show unique patterns for different emotional states. Hence, in this study, we focus on investigating the different emotional states using functional network analysis on different frequency bands. We construct multiple functional networks on different frequency bands and perform functional network analysis and time–frequency analysis on these frequency bands to find significant features representing the different emotional states. Furthermore, we also verify the effectiveness of these features by using them in emotion recognition.

## Materials and Methods

### Participants

The participants were selected from local native Chinese undergraduates or graduate students via interviews and survey questionnaires. Beck Anxiety Inventory (Grant, 2011), Hamilton Anxiety Rating Scale (Schneider et al., 2013), and Hamilton Rating Scale (Hamilton, 1986) were used to exclude individuals suffering from depression or other mental illness. Before beginning with the experiment, all participants were informed about the protocol, benefits, and risks of the study, and they signed an informed consent form. Finally, 24 healthy undergraduate students participated in this experiment (including 11 females), with a mean age of 22.3 years (range = 19–24, SD = 1.65). All participants were right-handed and had normal or corrected-to-normal vision. After the experiment, all the subjects were given a certain allowance for participating in the experiment.

### Stimuli

In this experiment, 180 pictures were selected from the Chinese Affective Picture System (CAPS) based on the normative valence and arousal ratings (Lu et al., 2005). The stimuli comprised 60 positive pictures (e.g., babies and flowers), 60 negative pictures (e.g., war scenes), and 60 neutral pictures (e.g., household objects). The normative ratings indicated that the stimuli had different valence degrees (positive: *M* = 6.85, SD = 0.25; neutral: *M* = 5.38, SD = 0.29; negative: *M* = 2.63, SD = 0.46) and different arousal degrees (positive: *M* = 5.35, SD = 0.44; neutral: *M* = 4.41, SD = 0.39; negative: *M* = 5.96, SD = 0.49). All pictures were displayed in the center against a black background on a 23-in computer screen with a refresh rate of 60 Hz. The subjects were seated ~70 cm from the computer screen during the experiment.

### Experimental Procedure

**Figure 1:**
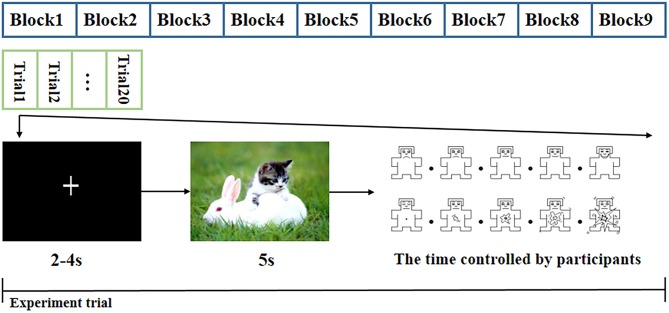
Experiment protocol.

The experiment began with a practice procedure to ensure that the subjects were familiar with the task. In this procedure, the subjects were presented with 10 additional CAPS pictures followed by the valence and arousal self-assessment Manikin rating scales (Morris, 1995). The pictures used in the practice procedure were different from the 180 pictures presented in the trials, and the EEG signals of the practice procedure were not used for the final data analysis. As shown in Figure 1, the formal experiment was divided into nine blocks. Each block included 20 pictures of the same category; two pictures of the same category did not appear in adjacent blocks. Each block comprised 20 trials, and the trials were displayed in a random order. A single trial began by displaying a “+” sign to draw the subject's attention to the subsequent picture. To avoid the anticipation effects associated with the display time of the “+” sign, the “+” sign was presented for 2–4 s in a random manner, and the data were used as the baseline. Then, one emotional picture was displayed for 5 s. Subsequently, the subject was rated on the valence and arousal rating scales, which were implemented by pressing any of the numeric keys between 1 and 9 on the keyboard within 10 s. A break was given between two blocks to alleviate the influence of the last block, and the basic break time was 2 min. The subjects could control the break time by pressing the button until they felt ready for the next block.

### Data Acquisition and Preprocessing

The experiment was performed in a professional electromagnetic shielding laboratory under suitable temperature and light conditions. In the experiment, the subjects sat in a comfortable chair at a distance of approximately 70 cm from the front screen. The size of the screen was 23 in with a refresh frequency of 60 Hz. EEG signals from 62 Ag/Ag-Cl scalp electrodes were continuously recorded using the g.HIamp System (g.tec Medical Engineering, Linz, Austria) with a sample rate of 512 Hz. The electrodes were positioned based on the 10–20 system. The Fz electrode and right earlobe were used as recording references, resulting in 61 effective electrodes. Online band-pass and notch filters were adopted for all channels to filter frequencies of 0.1–100 and 50 Hz, respectively.

Preprocessing procedures were performed to exclude artifacts and unrelated data. Epochs of 5,500 ms (500 ms before and 5,000 ms after the stimuli onset) were extracted from the raw EEG data of each picture. The mean voltage of the 500-ms segment before presenting the picture was subtracted as the baseline. Low frequency drift and high frequency noise were filtered out using a 0.1–80 Hz offline band-pass filter. Global artifacts were removed via average re-referencing. Additionally, we applied the Fast Independent Component Analysis (Fast ICA) algorithm (Hyvärinen, 1999) for blind-source analysis to remove electrooculography artifacts. Finally, a threshold of ±100 μ*v* was used to exclude artifacts with high amplitudes.

### Brain Functional Network Analysis in Different Frequency Bands

Following preprocessing, the artifact-free data were used to construct BNs for each data segment. Based on the 5,000-ms EEG data for each picture, we employed the coherence to measure the relationship between two electrodes. Coherence is an effective method commonly used to measure the function connectivity (Lee and Hsieh, 2014), and it estimates the linear relationship at a specific frequency between *x*(*t*) and *y*(*t*) of each pair of electrodes. Coherence is sensitive to the amplitude and phase changes, and its value ranges from 0 to 1. A high coherence implies that the signals from the two electrodes are working closely together. Coherence is denoted as follows:

(1) $$\begin{array}{r} C_{XY}\left(f\right)=\frac{{\left|P_{XY}\left(f\right)\right|}^{2}}{P_{XX}\left(f\right)P_{YY}\left(f\right)}, \end{array}$$

where *P*_*XY*_(*f*) is the cross-power spectral density (CPSD) estimate of *x*(*t*) and *y*(*t*) at the frequency*f*; *P*_*XX*_(*f*) and *P*_*YY*_(*f*) are the power spectral density (PSD) estimates of *x*(*t*) and *y*(*t*) at the frequency *f*, respectively. The PSD and CPSD values are calculated based on the p-welch method. *C*_*XY*_(*f*) is the coherence between *x*(*t*) and *y*(*t*) at frequency *f*. Then, the edge linkages were determined by averaging the coherence values within the five frequency bands: theta (4–8 Hz), alpha (8–13 Hz), beta (13–30 Hz), low gamma (30–50 Hz), and high gamma (50–80 Hz). The network of the delta frequency band (1–4 Hz) has not been discussed here because most of the EEG data did not have any effective coherence value under 4 Hz. Finally, we constructed five (one for each frequency band) 61 × 61 connectivity matrices for each stimulus.

According to graph theory, the functional networks of the brain can be effectively measured in terms of network properties (Straaten and Stam, 2013). To depict the BN, we computed the four basic BN properties: clustering coefficient (CC), characteristic path length (CPL), local efficiency (Le), and global efficiency (Ge) (Bassett and Bullmore, 2006; Jiang et al., 2009). CC and Le are measures to estimate the potential capacity of the local information processing. CC describes the degree of aggregation of the network nodes. Le is defined as the average efficiency of the local sub-graphs. CPL and Ge are used to determine the network potential for global information processing. CPL provides the values of the shortest path lengths between pairs of network nodes. Ge provides the capacity of the global information processing of the entire cerebral network. These network properties are defined by the following equations:

(2) $$\begin{array}{rcl} CC & = & {\frac{\underset{j,h\in \Theta }{\sum }\left(w_{ij}w_{ih}w_{jh}\right)}{\underset{j\in \Theta }{\sum }w_{ij}\left(\underset{j\in \Theta }{\sum }w_{ij}-1\right)}}^{1/3} \end{array}$$

(3) $$\begin{array}{rcl} CPL & = & \frac{1}{n}\underset{i\in \Theta }{\sum }\frac{\underset{i\in \Theta ,j\neq i}{\sum }d_{ij}}{n-1} \end{array}$$

(4) $$\begin{array}{rcl} Le & = & {\frac{\underset{j,h\in \Theta ,j\neq i}{\sum }\left(w_{ij}w_{ih}{\left[d_{jh}\left(\Theta_{i}\right)\right]}^{-1}\right)}{\underset{j\in \Theta }{\sum }w_{ij}\left(\underset{j\in \Theta }{\sum }w_{ij}-1\right)}}^{1/3} \end{array}$$

(5) $$\begin{array}{rcl} Ge & = & \frac{1}{n}{\underset{i\in \Theta }{\sum }\frac{\underset{j\in \Theta ,j\neq i}{\sum }\left(d_{ij}\right)}{n-1}}^{-1} \end{array}$$

### Emotion Recognition Based on Fusion Features

**Figure 2:**
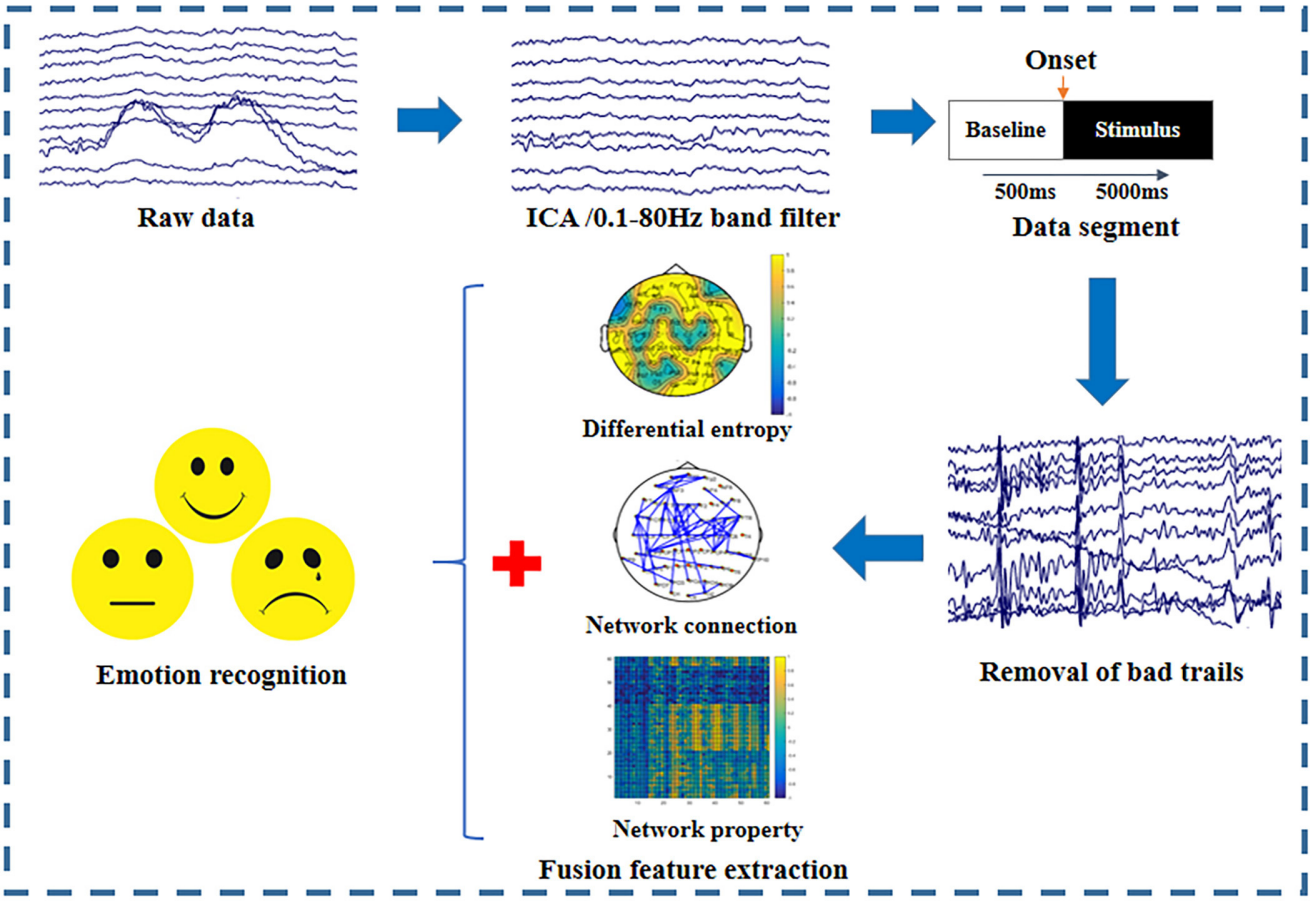
Emotion recognition procedures used in this experiment. Data preprocessing, fusion feature extraction, and pattern classification are the three main steps.

To investigate the effect of the EEG frequency bands on emotion recognition, we extracted multiple features in different frequency bands for further emotion classification. Multi-feature fusion could provide complementary information and improve the classification accuracy of emotion recognition. Therefore, we proposed to combine the network connections having significant differences among emotional states, BN properties, and differential entropies (DEs) in each frequency band as fusion features for emotion recognition. To remove the redundant information and eliminate the influence of different feature dimensions on the classification results, the maximum-relevance minimum-redundancy (MRMR) algorithm was used to select the top 61 features from the fusion features (Ding and Peng, 2005). Figure 2 depicts the entire emotion recognition procedure. First, the raw data were preprocessed to remove the artifacts; the preprocessing details are described in the section “Data Acquisition and Preprocessing.” Then, we extracted the top 61 important fusion features derived from the network connections with significant differences among the emotional states, BN properties, and DE.

For the DE feature, we used the short-time Fourier transform (STFT) to transform the preprocessed EEG data *s*(*t*)into the time–frequency domain. After performing STFT, we obtained the following relationship:

$$\begin{array}{r} STFT_{s,\gamma }\left(t,f\right)=\int_{-\infty }^{+\infty }s\left(\tau \right)\gamma^{*}\left(t-\tau \right)e^{-j2\pi f\tau }d\tau \\ \;=\int_{-\infty }^{+\infty }s\left(\tau \right){\gamma^{*}}_{t,f}e^{-j2\pi f\tau }. \end{array}$$

From *STFT*_*s*, γ_(*t, f*), we obtained the power of the theta (4–8 Hz), alpha (8–13 Hz), beta (13–30 Hz), low gamma (30–50 Hz), and high gamma (50–80 Hz) bands as follows:

(7) $$\begin{array}{r} spectrogram\left\{s\left[n\right]\right\}\left(m,f_{k}\right)={\left|S\left(m,f_{k}\right)\right|}^{2} \end{array}$$

DE is given as

(8) $$\begin{array}{r} DE=\log\left({\left|S\left(m,f_{k}\right)\right|}^{2}\right) \end{array}$$

The artifact-free EEG data were extracted from 61 channels; therefore, we acquired 61 DE features in each frequency band.

To reduce the computational complexity, all features were normalized to [0 1] by using the min–max normalization (MMN) method. MMN, a common method to normalize data, is given as follows:

(9) $$\begin{array}{r} X^{\prime }\left(i\right)=\frac{X\left(i\right)-Min}{Max-Min} \end{array}$$

where *X*(*i*) denotes a feature; *Min* and *Max* are the minimum and maximum values of all the features, respectively.

Finally, the fusion features were sent to the library for support vector machines (LIBSVM) for classification (Chang and Lin, 2011). To compare the performances of the fusion features in different frequency bands, we implemented LIBSVM with the linear kernel function and default parameter settings.

## Results

### SAM Ratings

As expected, the picture categories showed significant differences in both the valence and the arousal ratings. The valence and the arousal ratings of all subjects were averaged, and the rating scores of different stimulus groups were compared by *post-hoc* (paired *t*-test) analysis; the test results were corrected by using false discovery rate (FDR). The means and standard deviations of all subjects for the valence and the arousal ratings are shown in Table 1.

**Table 1:** Valence and arousal for positive, neutral, and negative pictures.

**Category**	***N***	**Valence *M* (SD)**	**Arousal *M* (SD)**
Positive	24	6.6 (0.27)	5.4 (0.39)
Neutral	24	5.2 (0.24)	3.9 (0.32)
Negative	24	2.8 (0.26)	6.2 (0.33)

For the valence rating scale, the positive pictures showed higher valence ratings than the neutral pictures (*p* < 0.05); the neutral pictures were rated as more positive than the negative pictures (*p* < 0.01). For the arousal rating scale, both the positive (*p* < 0.01) and the negative (*p* < 0.01) pictures achieved higher arousal ratings than the neutral pictures. The arousal ratings for the negative pictures (*p* < 0.05) were higher than those of the positive pictures.

### Neural Pattern Analysis in Different Frequency Bands

We statistically analyzed the neural patterns for different emotions using the following steps:

1. The BN connection values for each stimulus group were averaged for all subjects. Then, we implemented a one-way ANOVA with three factors (positive, neutral, and negative emotions) to test whether the BN connection strengths between the channels were significantly different for the positive, the neutral, and the negative emotions in the five frequency bands. The results were corrected using FDR. Our test results showed that several connections had significant differences (*p* < 0.001) in the BN, resulting in very dense connections, which were hard to observe in the network details. Therefore, we selected *P* = 1e10-12 as the threshold to display the most significant connections.
2. Four BN properties for each stimulus group were averaged for all subjects. The *post-hoc* test (paired *t*-test) was corrected using FDR correction. The test was used to analyze the differences of each BN property in the three groups being compared: (i) the group with significant differences in the BN properties between the positive and the neutral states, (ii) the group with significant differences in the BN properties between the positive and the negative states, and (iii) the group with significant differences in the BN properties between the negative and the neutral states.
3. Besides the differences in the connection patterns, we analyzed the scalp DE with significant differences among the three emotions. The DE values for the categories of stimuli were averaged for all the subjects. Then, the one-way ANOVA was corrected using FDR with three factors (positive, neutral, and negative emotions); this revealed whether the DE values of the same electrode were significantly different for the positive, the neutral, and the negative emotions in the five frequency bands.

**Figure 3:**
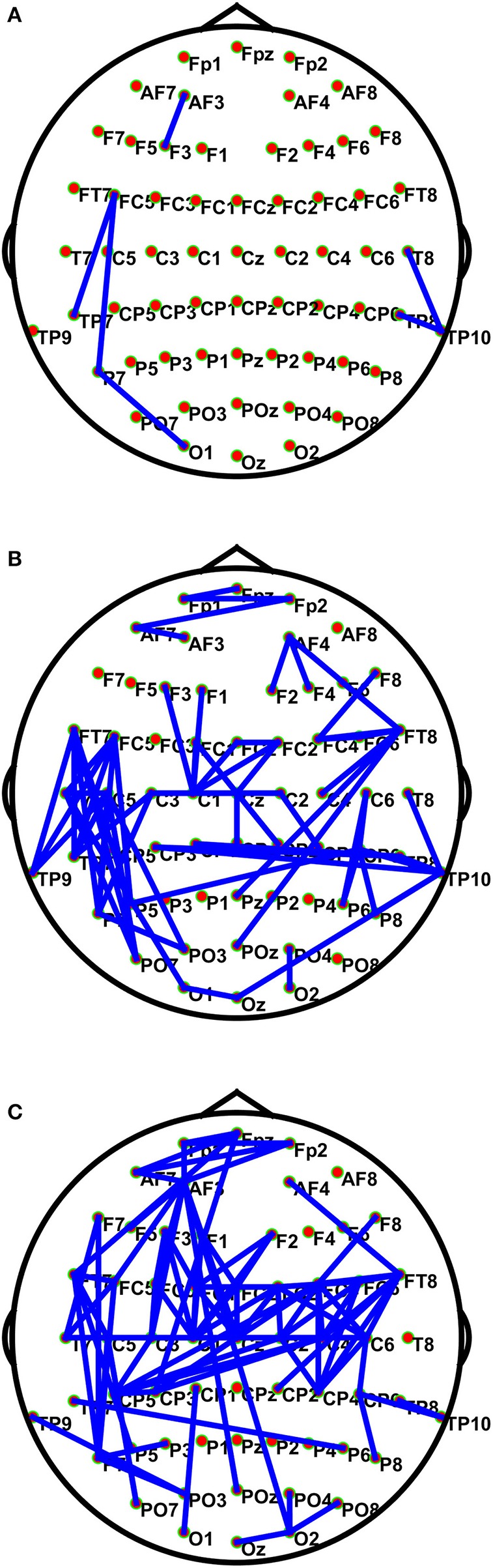
Connections with significant differences (*P* < 1e10-12) among the positive, neutral, and negative emotional states in different frequency bands. The subfigures **(A–C)** depict network connections with significant differences among the positive, neutral, and negative emotional states in the beta, low gamma, and high gamma bands, respectively.

Figure 3 shows the connections having significant differences (*P* < 1e10-12) among the positive, the neutral, and the negative emotions in different frequency bands. The BN connections with significant differences among emotions mainly occurred in the low and the high gamma bands. The connections in the high gamma band had significant left-side effects; the connection density of the left hemisphere was greater than that of the right hemisphere. Network connections with significant differences were mainly distributed in the left prefrontal lobe, the left temporal lobe, the parietal lobe, and the occipital region. There were long-distance connections in the high gamma band, which mainly existed between the prefrontal and the left temporal lobes (Fpz–FT7), the left temporal and the right parietal lobes (T7–C6), and the left temporal and the right occipital lobes (TP7–P6). Additionally, there were long-distance connections (AF3–O2 and AF3–POz) from the left prefrontal lobe to the occipital lobe across the whole brain. In the low gamma band, the left temporal lobe was much denser than the other brain regions. Network connections with significant differences were observed in the prefrontal lobe, the parietal lobe, and the right temporal lobe. Long-distance connections across the brain regions also occurred in this band, and they mainly existed in the left posterior frontal and the left occipital lobes (FT7–PO7), the left temporal and the left occipital lobes (C5–PO3), the right frontal and the occipital lobes (FT8–Pz and FT8–POZ), the right temporal lobe and the left parietal area (TP10–CP3), and the right temporal and occipital lobes (TP10–Oz). A few network connections exhibited significant differences in the beta band, and the linkages mainly existed in the temporal lobe. In the theta and the alpha bands, no network connections existed with significant differences among the emotional states.

In addition to analyzing the network connections with significant differences among the three emotions, we also investigated the significant differences in the BN properties between the two emotions in different frequency bands. Table 2 shows the *post-hoc* test (paired *t*-test) results of the BN properties in three comparison groups of different frequency bands. In Table 2, we can see that the differences in the BN properties for emotions were more significant in the high frequency bands than in the low frequency bands. In the high gamma band, the differences were most significant. In almost all bands, significant differences existed in the BN property between the positive and the neutral emotional states and between the negative and the neutral emotional states. However, significant differences could be observed between the positive and the negative emotional states only in the high gamma band. Ge could discriminate between the positive and the negative emotional states better than other BN properties, and it showed significant differences between the positive and the negative emotional states in the beta band, the low gamma band, and the high gamma band.

**Table 2:** Differences of brain network properties in three comparison groups of different bands.

**Frequency bands**	**Theta**			**Alpha**			**Beta**			**Low gamma**			**High gamma**		
**Groups**	**1**	**2**	**3**	**1**	**2**	**3**	**1**	**2**	**3**	**1**	**2**	**3**	**1**	**2**	**3**
CC	–	–	*	–	–	–	**	–	***	***	–	***	**	**	***
CPL	–	–	*	*	-	*	**	–	***	**	–	***	***	*	***
Le	–	*	*	*	-	*	**	–	***	***	–	***	***	**	***
Ge	–	–	-	**	*	–	*	***	*	***	*	***	***	*	***

*–, no significant difference*.

*P < 0.05; **P < 0.01; ***P < 0.001; 1, positive–neutral; 2, positive–negative; 3, negative–neutral.

**Figure 4:**
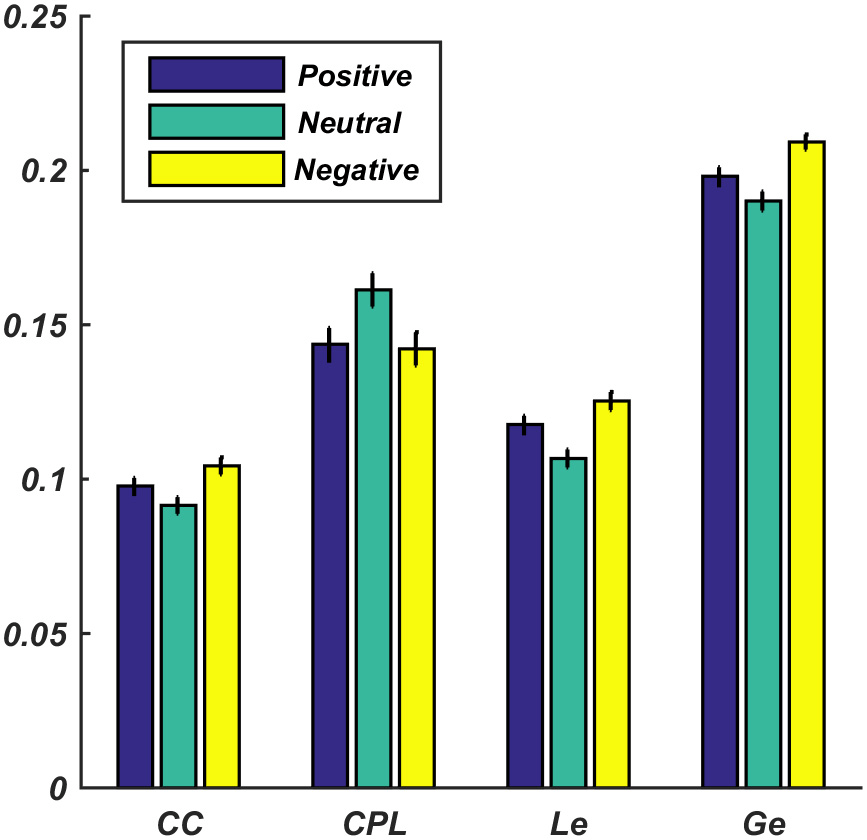
Average values for the four brain network properties in the high gamma band under positive, neutral, and negative emotional states. The error bars represent the standard deviations of the average values across all subjects.

Figure 4 shows all the subjects' average values of CC, CPL, Le, and Ge for different emotions in the high gamma band. The positive and the negative emotional states possessed a higher CC, Le, and Ge (*p* < 0.01) and a smaller CPL (*p* < 0.01) than the neutral emotional states. This might indicate that the information interaction rates of the brain under the positive and the negative emotional states were higher than the rate for the neutral emotional states. In addition, exposure to negative stimuli gave higher CC, Le, and Ge (*p* < 0.05) values than exposure to positive stimuli. However, the CPL (*p* < 0.05) value of the negative stimuli was less than that of the positive stimuli, i.e., the information interaction rate of the network for the negative stimuli was higher than that for the positive stimuli.

**Figure 5:**
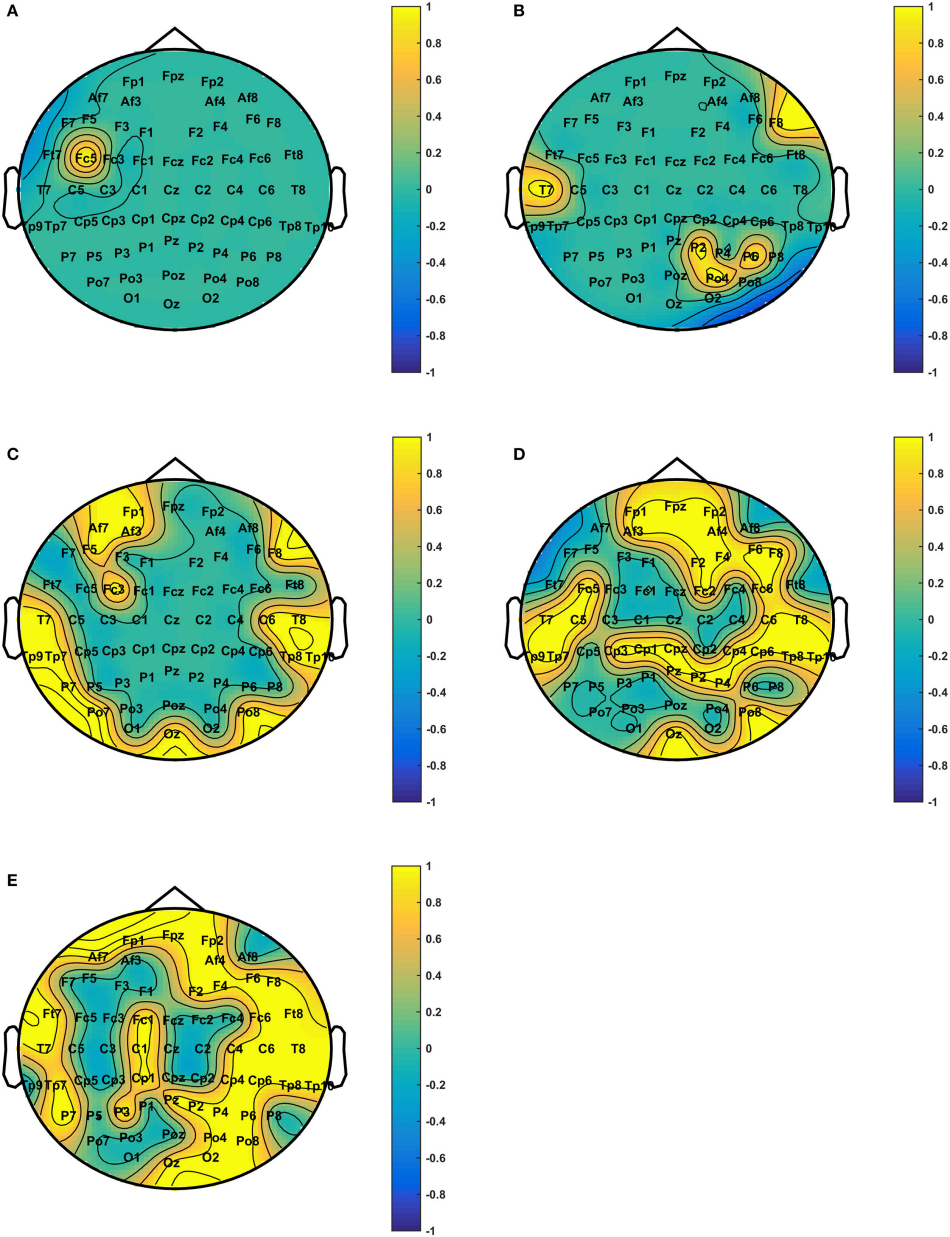
Scalp difference of the DE distribution among positive, neutral, and negative emotional states in different frequency bands. The yellow electrodes denote DE with statistically significant differences (*P* < 0.01) among the three emotional states. The subfigures **(A–E)** depict the *DE* distribution with significant differences among the positive, neutral, and negative emotional states in the theta, alpha, beta, low gamma, and high gamma bands.

After analyzing the network connection patterns, we analyzed the topological differences of the DE distributions under various emotional states in different frequency bands. Figure 5 shows the DE distribution with significant differences (*p* < 0.01) among the three emotional states in each frequency band. We discovered that the DE distribution with significant differences mainly existed in the high frequency bands, and most of the electrodes with significant differences were in the high gamma band. These electrodes were mainly distributed on the prefrontal lobe, the bilateral temporal lobe, the parietal lobe, and the right occipital region.

### Emotion Classification Results on Our Dataset and Public Dataset

Apart from the statistical analysis, the performances of features under different frequency bands were compared in the emotion recognition experiment. The emotion classification procedures are shown in Figure 2. Based on the results of the statistical analysis, we combined the network connections that showed significant differences for the three emotional states, DE, and Ge in each frequency band, and we formed fusion features. The top 61 important features were selected in each frequency band via MRMR for the final emotion classification.

**Figure 6:**
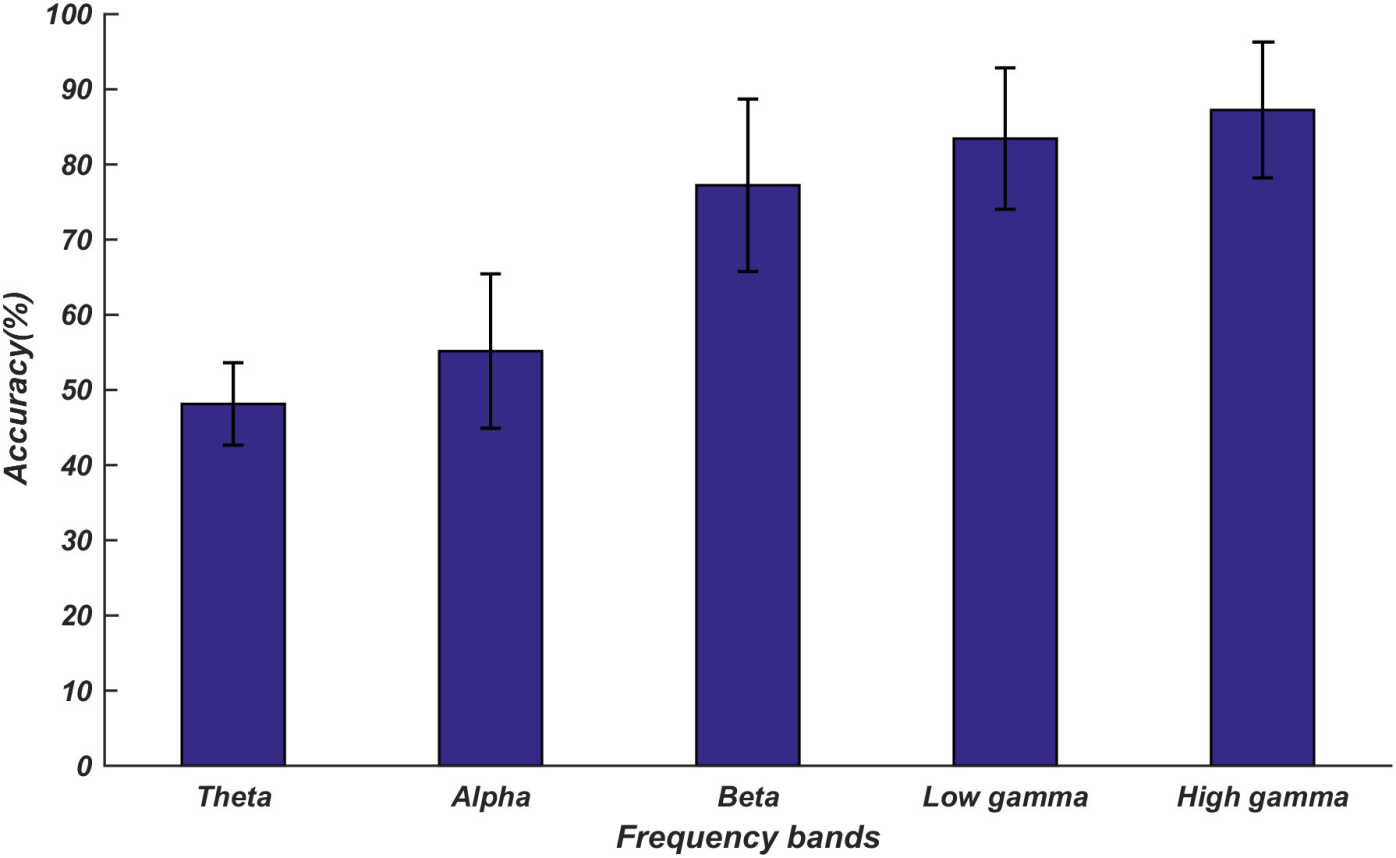
Average accuracies for the classification of emotions into three categories based on the proposed fusion features. The five accuracy bars represent the average accuracies (48.14, 56.83, 78.36, 83.44, and 87.27%) in different frequency bands. The error bars represent the standard deviations of the average accuracies across all subjects.

The classification results are shown in Figure 6. The highest classification accuracy of 87.27% was obtained for the high gamma band fusion features. The features in the high frequency bands performed better than the features in the low frequency bands; this was consistent with the results of the above-described statistical analysis.

We further investigated the classification performances of the proposed fusion features in different frequency bands on Zhuang's dataset (Zhuang et al., 2018). This dataset includes emotional EEG signals recorded for 30 healthy subjects. During the experiment, the subjects watched 18 movie clips with six emotion tags: joy, neutrality, sadness, disgust, anger, and fear. After watching each movie clip, the subjects were asked to recall a scene from the movie to self-elicit emotion. Therefore, each subject recorded two types of EEG data: movie clip-elicited and self-elicited. In this work, we only use the movie clip-elicited EEG signals. The EEG signals were recorded using g.HIamp System at a sampling rate of 512 Hz from 62 electrodes positioned based on the 10–20 system. The emotion recognition procedures were similar to those shown in Figure 2. For the preprocessing of the EEG data (following Zhuang's work), we first extracted the last 50 EEG signals of each movie clip. Then, we used the 0.1–80 Hz band-pass filter, Fast ICA, and baseline correction to remove the noise and the artifacts. For each subject, the preprocessed data were segmented into 882 samples using a 2-s window with half overlap between two consecutive windows. Then, the coherence functional network connection, DE, and Ge were calculated in the five frequency bands, and these features were combined as fusion features. Finally, the top 61 important fusion features selected by MRMR in each frequency band were sent into LIBSVM to classify the emotions into the abovementioned six categories.

**Figure 7:**
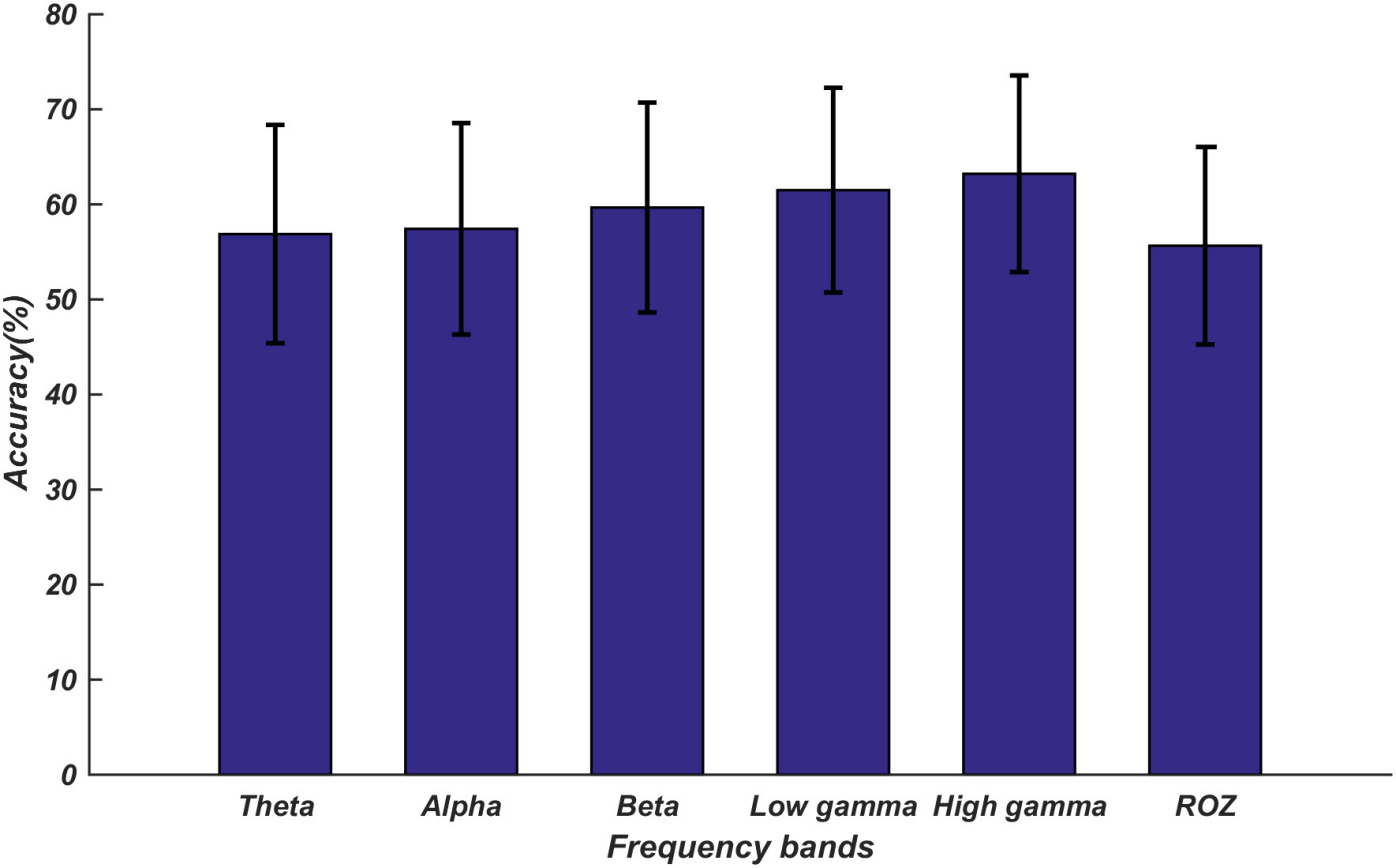
Average accuracies for the classification of emotions into six categories on the public dataset. The error bars represent the standard deviations of the average accuracies across all subjects. The top five accuracy bars represent the average accuracies (56.23, 57.43, 59.15, 61.28, and 63.36%) of the proposed fusion feature in different frequency bands. The last accuracy bar named ROZ represents the average accuracy (55.65%) in Zhuang's study.

The classification results are displayed in Figure 7. We found that these classification results were consistent with the results obtained for our dataset; the features of the high gamma band still achieved the highest accuracy. In addition, the fusion feature used in our current work was superior to the features extracted by Zhuang; the accuracy of the high gamma band fusion feature was nearly 7% higher (*p* < 0.01) than that in Zhuang's research (Zhuang et al., 2018).

In each frequency band, the fusion feature had 183 dimensions, which included 61 dimensions each of brain network connections, Ge, and DE. We selected the top 61 features from the 183 fusion features via MRMR to classify the emotions into six discrete categories. The most selected 30 features in the high gamma band are shown in Table 3.

**Table 3:** Most selected features by maximum-relevance minimum-redundancy in the high gamma band.

**Feature types**	**Selected features**
Ge	T7, TP8, PO8, TP10
DE	Fp2, FC6, FT8, T7, C5, T8, TP8, Oz, TP9, TP10
BNC	Fpz-Fp2, Fpz-AF4, Fpz-Fp1, Fpz-AF7, Fp2-AF4, Fp2-Fp1, AF4-F4, AF3-F5, F7-FC5, F7-T7, F7-F5, F1-FCz, F6-F8, F8-FC6, FC1-C1, FC4-FT8

## Discussion

### Neural Signature of Emotional States in the High Gamma Band

High-frequency EEG signals have been widely used for studying advanced cognitive functions such as emotions. In this research, we analyzed the network connections having significant differences and the potential relationships between the BN properties and emotions. We also investigated the topographical differences of the DE distribution among emotional states in five frequency bands. Our results proved that the high-gamma-band EEG signals were more closely related to the emotional states.

Connections in the high frequency band have been reported to be important for the processing of emotional states (Flores-Gutiérrez et al., 2007; Hossein and Sahar, 2016). In this study, we observed that connections with significant differences among the positive, the neutral, and the negative emotions were denser in the high gamma band than in the low gamma band. These denser connections with significant differences between the emotional states in the high frequency bands may indicate that high frequency components in EEG could mediate information transmission when the brain concentrates on processing the emotion-related activities (Li et al., 2019).

Further, connections with significant differences among the positive, the neutral, and the negative emotions exhibited left-side effects. The different connections mainly occurred in the left prefrontal, the left temporal, the parietal, and the occipital regions. Left hemisphere activation has been associated with positive emotions and right hemisphere activation has been related to negative emotions in the literature (Müller et al., 1999; Flores-Gutiérrez et al., 2007). Costa et al. also reported that sadness is associated with a wider synchronization between the right and the left frontal sites within the left hemisphere (Costa et al., 2006). The connection patterns observed in this experiment further demonstrated the existence of side effects in the brain when dealing with different emotions.

Interestingly, long-distance connections exist with significant differences among the emotional states of the various brain regions (Fpz–FT7, T7–C6, TP7–P6, and AF3–Cz); such differences are even observed among the long-distance connections across the whole brain regions (AF3–O2 and AF3-POz) in the high gamma band. Long-distance connections in the high frequency band have been reported to be associated with complex cognition activities. Rodriguez et al. analyzed Mooney face-induced EEG network connections in the gamma band (30–80 Hz) and discovered synchronized and asynchronous connections between the parietal, the occipital–temporal, and the frontal–temporal lobes; they believed that the long-range character of phase asynchrony may be a mechanism that subserves large-scale cognitive integration (Rodriguez et al., 1999). High-order long-range phase synchrony was also observed between the anterior delta and the posterior gamma oscillations in musicians when they listened to music (Bhattacharya and Petsche, 2005). Emotion is a complex cognitive activity; these highly integrated long-distance connections may indicate that a functional cooperation exists between the frontal and the occipital lobes, which can improve the information integration efficiency of the brain (Flores-Gutiérrez et al., 2007). Studies have proposed that the visual attention involved in watching emotional pictures is controlled by both top–down cognitive factors and bottom–up sensory factors. These two types of process mechanisms are reflected in the frontal–parietal, the temporal–parietal, and the parietal–occipital activities (Desimone and Duncan, 1995; Clark and Hillyard, 1996; Li et al., 2010; Müsch et al., 2017). Therefore, long-distance connections among the frontal, the parietal, the temporal, and the occipital lobes may reflect the bottom–up and top–down mechanisms during affective stimuli processing.

The BN properties in almost all frequency bands showed significant differences between the neutral emotional state and the positive and the negative emotional states. However, for low frequency band BN properties, no significant difference was observed between the positive and the negative emotions. In the high gamma band, there were significant differences (*p* < 0.05) of BN properties between the positive and the negative emotional states. These results indicated that the positive and the negative emotional states shared the same BN connection pattern or there was similar network efficiency in the low frequency bands. In the high gamma band, more differences existed between the positive and the negative emotional states (Lindquist et al., 2016).

Consistent with the results of network connections, the DE distribution differences among the emotional states mainly occurred in the high gamma band. The differences mainly existed in the frontal, the temporal, the parietal, and the right occipital lobes, which were consistent with the results of Zhuang et al. (2018) who observed that DE from the channels located on the frontal, the temporal, and the occipital lobes exhibits more significant differences among the emotional states. These results further demonstrate that the high gamma components in EEG are associated with emotion processing. Differences exist in the DE distribution in multiple brain regions, which also demonstrates that emotional activity requires the cooperation of multiple related brain regions (Bassett and Bullmore, 2006; Zhang et al., 2017).

### Relationships Between BN Properties and Emotion Arousal

The potential relationship between the BN properties and stimuli arousal in different frequency bands was also investigated. As shown in Table 4, in each frequency band, all BN properties were significantly related to picture arousals (*p* < 0.01), and in the high gamma band, the relationship was most significant. In addition, CC, Le, and Ge were positively correlated (*p* < 0.01) with the arousal, whereas CPL was negatively correlated (*p* < 0.01) with the arousal. This showed that the CC, Le, and Ge values were higher, and the CPL value was smaller for high arousal rating stimuli as compared with low arousal stimuli. The high CC, Le, and Ge values and the small CPL value represented the high information processing efficiency of the brain (Li et al., 2015). In addition, Figure 3 shows the connections with significant differences (*P* < 1e10-12) among the positive, the neutral, and the negative emotional states in different frequency bands. The subfigures (A), (B), and (C) depict network connections with significant differences among the positive, the neutral, and the negative emotional states in the beta, the low gamma, and the high gamma bands, respectively.

**Table 4:** Pearson correlation coefficients between the brain network properties and picture arousal in different frequency bands.

**BN property**	**Theta**	**Alpha**	**Beta**	**Low gamma**	**High gamma**
CC	*R* = 0.278*	*R* = 0.354*	*R* = 0.536**	*R* = 0.608**	*R* = 0.588**
CPL	*R* = −0.265*	*R* = −0.25*	*R* = −0.586**	*R* = −0.602**	*R* = −0.638**
Le	*R* = 0.294*	*R* = 0.314*	*R* = 0.565**	*R* = 0.611**	*R* = 0.624**
Ge	*R* = 0.216*	*R* = 0.226*	*R* = 0.513**	*R* = 0.462*	*R* = 0.603**

* *P < 0.05*;

** *P < 0.01*.

The average CC, Le, and Ge values in the high gamma band for the positive and the negative stimuli were higher than those for the neutral stimuli, whereas the average CPL value for the positive and the negative stimuli was smaller than that for the neutral stimuli. These results further demonstrate that the BNs of the positive and the negative stimuli were more efficient than those for the neutral stimulus (Zhang et al., 2017). Furthermore, the CC, Le, and Ge values for the negative pictures were higher (*p* < 0.01) than those for the positive picture; the negative pictures had a smaller (*p* < 0.01) CPL value than the positive pictures. This proves that the brain was more active and more efficient in responding to the negative stimuli than to the positive ones. Other researchers have also reported that negative emotional activity required more attention, and the cortical activity was enhanced when the brain responded to negative stimuli (Ning, 2012; Jin et al., 2014; Ding et al., 2017).

### Superiority of High Gamma Band Features in Emotion Recognition

The classification results demonstrated that high gamma band features were more effective in emotion recognition. Our proposed fusion features received the highest classification accuracy in the high gamma band for both our dataset and the public dataset. The results obtained for emotion recognition are consistent with the neural signature analysis results, which state that features in the high gamma band can more effectively reflect the differences between various emotions than features in the low gamma band. On the public dataset, we obtained the highest accuracy of 63.36% with high gamma band fusion features, which was 7% higher than Zhuang's results (Zhuang et al., 2018). The classification results also demonstrated that the features of the high gamma band EEG were superior to those in the other frequency bands. The classification improvement on the public dataset can be attributed to the complementary information of fusion features. The results in Table 3 show that the fusion features selected by MRMR include the compensative features derived from the brain function network and DE. The BN connections and Ge could reflect the information transmission and processing of the whole brain. DE represented the activation patterns of each channel while the brain processed the emotional activity. Therefore, the fusion feature has the advantage of involving a single feature that provides complementary information for emotion recognition. The public dataset included EEG signals recoded under six discrete emotional states; these discrete emotions were different in both the valence and the arousal dimensions (Dan, 2012). The statistical analysis results showed that the BN properties were significantly related (*p* < 0.05) to arousal and showed significant differences (*p* < 0.05) among the different valence ratings of emotions in the high frequency bands. Therefore, the fusion feature could discriminate among emotions not only from the valence dimension but also from the arousal dimension. Another point is that we selected only Ge from the four BN properties as a component of fusion feature for emotion recognition. According to the graph theory, Ge represents the information integration and the exchange efficiency of the whole network (Li et al., 2016). The statistical results showed that Ge was more sensitive than other BN properties in depicting the differences among the different emotional states. Ge was more effective in emotion recognition. In addition, network connections were proposed as a feature for emotion recognition, and the classification results demonstrated that network connections were effective features for emotion recognition. In short, the features extracted based on BN are effective and crucial for emotion recognition.

Previous event-related potential research have reported that significant differences of EEG signals among emotional states can be observed in P2 and P3 and slow-wave time windows (Ding et al., 2017; Wang and Li, 2017). However, the network connections and information processing and propagation in these time windows remain unclear. In the future, we will construct time-varying BNs (Li et al., 2016) and investigate the neural mechanism of the brain in more precise time windows.

## Conclusion

In this research, we investigate the network connection patterns under different emotional states in the high gamma band (50–80 Hz). Functional BNs were constructed in different frequency bands based on EEG signals induced by positive, neutral, and negative pictures. We performed network connection and time–frequency analysis on different frequency bands to determine the significant features representing different emotional states. The results showed that the network connections in high frequency bands with significant differences among the positive, the neutral, and the negative emotional states showed left-side effect, and these networks were much denser than the network connections in other frequency bands. Long-distance connections with significant differences among the emotional states were also observed in the high frequency bands. Additionally, high gamma band fusion features derived from Ge, network connections, and DE achieved the highest classification accuracies on both our dataset and the public dataset. These results provide further evidence that high-gamma-band signals are more sensitive and effective in emotion analysis than low-frequency signals.

## Data Availability Statement

The datasets generated for this study are available on request to the corresponding author.

## Ethics Statement

The studies involving human participants were reviewed and approved by the ethical committee of Henan Provincial People's Hospital. The patients/participants provided their written informed consent to participate in this study.

## Author Contributions

KY was mainly responsible for research design, data collection, data analysis, and manuscript writing of this study. LT was mainly responsible for research design and data analysis. JS was mainly responsible for data collection and production of charts. NZ was mainly responsible for data analysis and document retrieval. YZ was mainly responsible for data analysis and manuscript writing. BY was mainly responsible for research design and manuscript writing.

### Conflict of Interest

The authors declare that the research was conducted in the absence of any commercial or financial relationships that could be construed as a potential conflict of interest.
